# Exploring the Connection Between the Structure and Activity of Lignin-Derived Porous Carbon Across Various Electrolytic Environments

**DOI:** 10.3390/molecules30030494

**Published:** 2025-01-23

**Authors:** Zhihao Ding, Rui Wang, Guoli Pang, Lili Dong, Tingzhou Lei, Suxia Ren

**Affiliations:** 1 School of Environmental Science and Engineering, Changzhou University, Changzhou 213164, China; s22030857009@smail.cczu.edu.cn (Z.D.);; 2 Nation-Local Joint Engineering Research Center of Biomass Refining and High-Quality Utilization, Institute of Urban and Rural Mining, Changzhou University, Changzhou 213164, China; 3 School of Petrochemical Engineering, Changzhou University, Changzhou 213164, China

**Keywords:** lignin, supercapacitor, porous carbon, electrochemical properties, electrode materials

## Abstract

Porous carbon holds great potential for application in supercapacitors due to its rich pore structure and high specific surface area. In this research, lignin served as the starting material for the production of lignin-derived carbon materials via a carbonization-activation process. The resulting porous carbon materials underwent rigorous characterization using SEM, BET, Raman, XRD, and XPS to uncover their morphological and structural intricacies. Notably, the optimal product, achieved with a mass ratio of lignin to KOH and KCl at 1:2:0.5 and activation temperature at 700 °C, emerges as an excellent electrode material for high-performance supercapacitors. This superior carbon material boasts a remarkable specific surface area of 2730 m^2^ g^−1^, demonstrating an electrochemical capacitance up to 406 F/g at 1 A/g, its high performance surpasses many existing carbon materials. To further investigate the potential application of ELC in electric double-layer capacitors, the electrochemical properties of ELC in 6 M KOH, 1 M Na_2_SO_4_, and 1 M Et_4_NBF_4_/PC electrolytes were investigated, the reasons for the differences in ELC’s electrochemical performance in different electrolytes are discussed and analyzed in detail, and the advantages and disadvantages of ELC’s performance in capacitor devices of different systems are compared and analyzed. This was performed to compare the electrochemical performance of ELC and commercial YP-50F capacitor carbon in an electric double-layer capacitor, and to investigate the potential application of ELC.

## 1. Introduction

With the increase in global energy demand and the increase in environmental protection awareness, there is a growing international consensus and urgent need to explore and utilize sustainable and environmental protection new energy technology [1]. The development of clean energy heavily relies on advanced electrochemical energy storage and conversion systems, including lithium-ion batteries [2], lithium-silicon batteries [3], lithium-selenium batteries [4], in-plane micro batteries [5], etc. In this context, electric energy, recognized for its cleanliness and efficiency, necessitates advancements in storage and utilization technologies. This development is linked to the enhancement of energy storage devices, such as batteries and supercapacitors, with a focus on improving performance and reducing costs. Supercapacitors, especially electric double-layer supercapacitors (EDLCs), exhibit considerable potential for application in energy storage due to their fast charge–discharge capabilities, high power output, and long cycle stability [6]. The performance of supercapacitors is critically influenced by the properties of electrode materials, such as specific surface area, pore structure, conductivity, etc.

Despite the development of various high-performance electrode materials, including activated carbon, graphene, and carbon nanotubes, significant challenges related to cost, sustainability, and the feasibility of large-scale production hinder their widespread application. Therefore, the pursuit of low-cost, high-performance, and environmentally friendly electrode materials has emerged as a critical focus for researchers. Among the various materials under consideration, lignin-derived carbon composites have attracted significant interest owing to their advantageous properties. These properties include a rich pore structure, exceptional conductivity, and affordability [7]. Carbon-based materials, in general, exhibit excellent electrical conductivity, a hierarchical pore structure, and remarkable electrochemical stability. However, a major limitation of most carbon precursors is their unsuitability for large-scale synthesis, primarily due to high costs and environmental pollution concerns. Porous carbon materials are frequently employed as carrier materials due to their high specific surface area, high electrical conductivity, and good stability [8]. Lignin, which is one of the primary components of plant cell walls and is derived from a diverse range of sources, represents the second most abundant organic substance in nature. Through processes such as pyrolysis and activation, lignin can be transformed into carbon materials with a high specific surface area and a graded pore structure. This makes lignin a highly promising candidate for use in electrodes for EDLCs. In commercial supercapacitors, activated carbon electrodes are widely used due to their exceptional conductivity, large surface area, chemical stability, and low cost [9]. Thus, the potential of lignin-derived carbon composites to meet these criteria further underscores their significance in the field of supercapacitor technology.

The pore structure, surface chemical properties, and electrochemical performance of lignin-based carbon materials are influenced by the selection of raw materials, pretreatment methods, and carbonization and activation conditions. Various sources of lignin, including wood, agricultural residues, and black liquor, along with distinct processing methodologies, yield carbon materials with unique pore structures and surface attributes, which subsequently impact their electrochemical behavior across different electrolyte systems. For example, the specific surface area, pore size distribution, and surface functional groups of lignin-derived carbon can be regulated by optimizing the carbonization temperature and the choice of activator, thus enhancing their charge storage capacity in a specific electrolyte. Previous studies have demonstrated that lignin-based porous carbon (ELC) exhibits excellent electrochemical properties in the KOH electrolyte, highlighting its potential as an EDLC electrode material. Therefore, it is very important to explore the application prospect of lignin-based porous carbon in alternative electrolyte systems, as well as to further examine how the structure of lignin-based porous carbon affects its electrochemical performance in different electrolytes.

Therefore, the aim of this study is to investigate the relationship between the microstructure and electrochemical properties of lignin-based carbon materials, especially in different systems such as Na_2_SO_4_, KOH, and organic electrolytes such as Et_4_NBF_4_/PC. Through systematic electrochemical testing and characterization, the fundamental reason of the difference in capacitance performance of lignin-based carbon materials in different electrolytes was revealed. Additionally, a correlation model linking the structure and performance of the materials has been developed. This study not only helps to deepen the understanding of the electrochemical behavior of lignin-based carbon materials but also provides a scientific foundation for the design and optimization of lignin-based supercapacitors, thereby facilitating the high-value utilization of lignin as a renewable resource in the field of high-performance energy storage.

## 2. Results and Discussion

### 2.1. Morphologies and Structural Characterization of Lignin-Derived Activated Carbon

**Figure 1:**
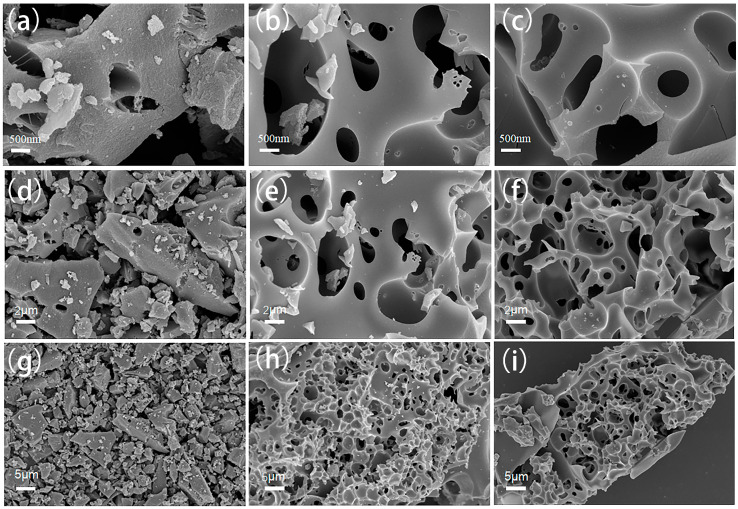
SEM images of YP-50F (**a**,**d**,**g**), ELC-KOH (**b**,**e**,**h**), and ELC-KOH-KCl (**c**,**f**,**i**).

The microstructure of the prepared carbon was observed by SEM. As shown in Figure 1, the YP-50F sample exhibits a smooth and even surface morphology. In contrast, the ELC-KOH and ELC-KOH-KCl samples display a distinct void structure. The results indicate that the KOH activation process has a good etching effect on ELC porous carbon materials, leading to alterations in their surface architecture. Further observation reveals that that the introduction of KCl during the activation process results in an increase in the number of pores on the material’s surface. These modifications not only enhance pore density but also improve the connectivity among the pores, culminating in the formation of a three-dimensional cross-linked network structure within the sample. The three-dimensional cross-linked network architecture of ELC facilitates the presence of active sites for ion adsorption while simultaneously creating more efficient pathways for the rapid transfer of electrolyte ions. This design minimizes the ion transport distance within micropores and mitigates the issue of micropore obstruction caused by ion accumulation, thereby enhancing the efficiency of ion adsorption. Such improvements are crucial for attaining rapid ion transfer in supercapacitor applications [10].

**Figure 2:**
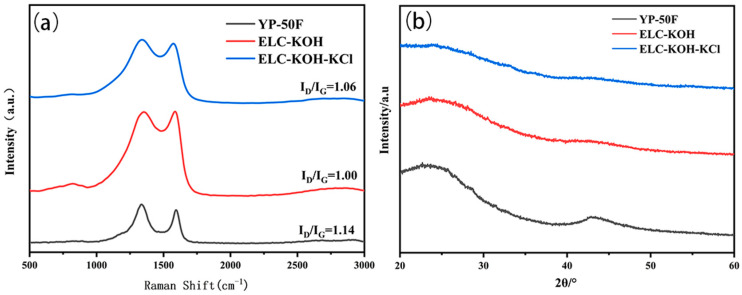
Raman spectra (**a**) and XRD (**b**) plots of YP-50F, ELC-KOH, and ELC-KOH-KCl.

The results of Raman and XRD are presented in Figure 2. In the Raman spectra (Figure 2a), the D-band at 1349 cm^−1^ and the G-band at 1580 cm^−1^ are indicative of structural defects and distortions, as well as the in-plane vibration and graphitization of SP_2_-hybrid carbon atoms, respectively [11]. The ratio of the intensities of the D-band to the G-band (I_D_/I_G_) suggests the degree of defects within the carbon material. The I_D_/I_G_ values of the YP-50F, ELC-KOH, and ELC-KOH-KCl samples were 1.14, 1.00, and 1.06, respectively, which indicated that these samples exhibit amorphous characteristics and a significant presence of defects. The XRD patterns of the porous carbon reveal two broad diffraction peaks at 26.3°and 43.6° which correspond to the (002) and (100) plane reflections of the disordered carbon layer, respectively. The addition of KCl resulted in an increase in the width of the (100) plane diffraction peak for the ELC-KOH-KCl material, indicating that the synergistic etching of KOH and KCl promoted the formation of more pores or defects [12]. Consequently, this led to a reduction in the ordering degree of ELC-KOH-KCl, which is consistent with the results of Raman. The presence of pore defects in carbon materials has the potential to enhance the number of active sites on their surfaces, thereby augmenting their adsorption characteristics. Additionally, these defects can lead to an increase in the specific surface area and the development of a microporous structure within the carbon materials. This modification can enhance ion transport capabilities and alter the electronic structure of the carbon materials, resulting in improved conductivity. Such enhancements are particularly advantageous for optimizing the electrochemical performance of supercapacitor electrodes [13].

**Figure 3:**
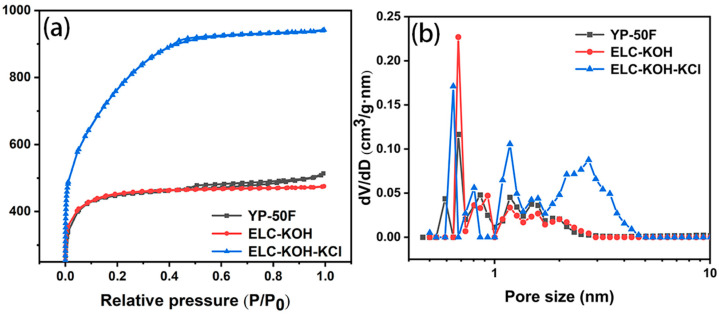
N_2_ adsorption/desorption isotherms for YP-50F, ELC-KOH, and ELC-KOH-KCl samples (**a**) and pore size distribution curves (**b**).

The specific surface area and pore size distribution of the materials were determined by measuring the N_2_ adsorption isotherms of the three samples. The results are shown in Figure 3. As can be seen from the diagram, YP-50F, ELC-KOH, and ELC-KOH-KCl all exhibit a mixed Sorption isotherm of type I and type IV. In the region of low relative pressure (P/P_0_ < 0.4), there is a marked increase in the adsorption volume, indicating the existence of a large number of micropores in the material. Hysteresis loops were observed in YP-50F and ELC-KOH-KCl in the middle pressure region (0.4 < P/P_0_ < 0.9), indicating the presence of mesopores in these samples. The specific surface area and total pore volume of commercial activated carbon YP-50F were 1700 m^2^·g^−1^ and 0.77 cm^3^·g^−1^, respectively (Table 1). The specific surface area of ELC-KOH was 1670 m^2^·g^−1^, with the pore size distribution predominantly within the range of 0.35–2 nm. In contrast, ELC-KOH-KCl exhibited a pore size distribution primarily between 0.35–10 nm, which differs from that of ELC-KOH. The specific surface area of ELC-KOH-KCl is 2730 m^2^·g^−1^, and the total pore capacity is 1.45 cm^3^·g^−1^. The high specific surface area and good pore size distribution are beneficial to the high capacitance and power performance of ELC supercapacitors.

**Figure 4:**
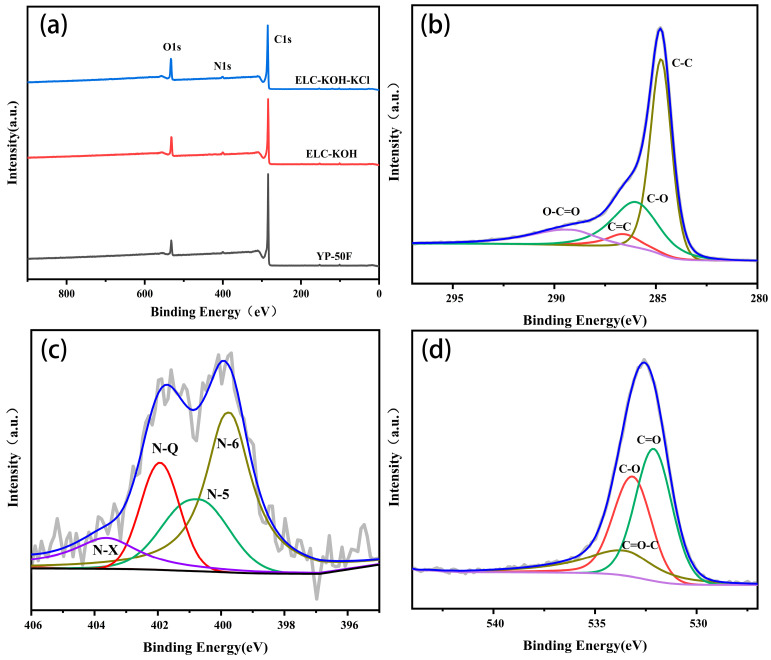
XPS survey spectra of YP-50F, ELC-KOH, ELC-KOH-KCl (**a**), and (**b**) C1s, (**c**) N1s, (**d**) O1s of sample ELC-KOH-KCl.

The elemental composition and surface chemical properties of the samples were studied by XPS. As shown in Figure 4a, it can be seen that the sample comprised only C, N, and O. Figure 4b presents the core level spectra of the C1s region for the PLC-KOH sample, which were deconvoluted into four distinct peaks. The main peak, observed at 284.8 eV, corresponds to graphitic carbon (C–C), while additional peaks are identified at 285.7 eV(C-O), 285.9 eV(C=C), and 289.5 eV(O-C=O) [14]. The presence of oxygen in the spectrum is suitable for wettability during the electrode fabrication process.

The high-resolution of N1s spectra (Figure 4c) are deconvoluted into four peaks located at 399.3, 400.6, 401.3, and 403.5 eV, correspond to the following nitrogen functionalities: pyridinic (N-6), pyrrolic (N-5), graphitic (N-Q), and N-oxide (N–X) [15]. In addition, the high-resolution O1s spectra (Figure 4d) demonstrate that the ELC-KOH-KCl presents mainly three peaks associated with C=O, C–O, and C=O-C groups at binding energies of 532.0, 533.2, and 533.7 eV, respectively. The presence of these oxygen-containing functional groups enhances wettability, thus extending the ion-accessible surface area available for charge storage [16]. Furthermore, the incorporation of N-6 and N-5 introduces various defects that create additional active sites for redox reactions, while graphite nitrogen enhances electron transport, thereby increasing the electrical conductivity of the carbon material [17]. Current research indicates that the incorporation of O and N functional groups can enhance the surface characteristics of electrode materials. This enhancement leads to a significant increase in the effective contact area between electrolyte ions and electrode materials. Additionally, the formation of surface functional groups improves hydrophilicity in KOH solution, thereby contributing to improve the capacitance performance of supercapacitors [18,19].

### 2.2. Electrochemical Performance

**Figure 5:**
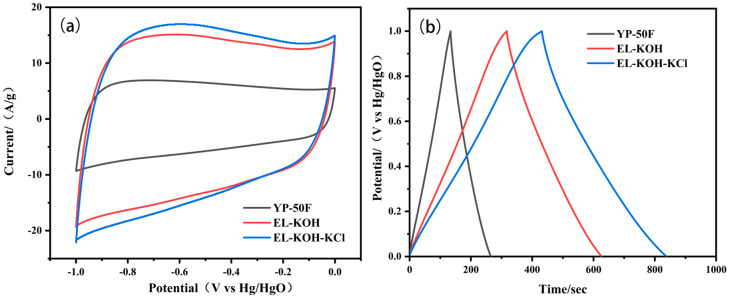
CV curves of YP-50F and ELC electrodes at a scan rate of 50 mV s^−1^ (**a**); GCD curves of YP-50F, ELC electrode at 1 A g^−1^ in 6 M KOH electrolyte (**b**).

The capacitance behavior of ELC and YP-50F electrodes in Na_2_SO_4_ and KOH aqueous electrolytes were evaluated utilizing using a three-electrode system. As depicted in Figure 5a, the CV curve of the carbon material in the 6 M KOH electrolyte exhibits a rectangular shape, indicating that the main mechanism of energy storage is double-layer capacitance. The integral area of the CV curve for ELC-KOH-KCl is the largest, indicating that it possesses the highest specific capacitance. The GCD presented in Figure 5b shows a similar isosceles triangle shape, further confirming the energy storage mechanism of the double-layer capacitor and demonstrating its favorable capacitance performance. The specific capacitance of YP-50F, ELC-KOH, and ELC-KOH-KCl are 131.3, 309.3, and 406.3 F g^−1^, respectively. ELC-KOH-KCl material exhibits the best electrochemical performance, which can be attributed to its high specific surface area and abundant multistage pore structure that facilitates rapid ion transport within the electrolyte.

**Figure 6:**
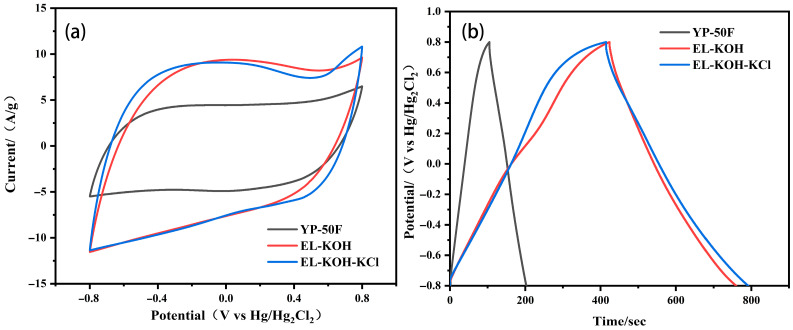
CV curves of YP-50F and ELC electrodes at 50 mV s^−1^ (**a**); GCD curves of YP-50F and ELC electrode at 1 A g^−1^ in 1 M Na_2_SO_4_ electrolytes (**b**).

The electrochemical properties of the prepared carbon material at 1 m Na_2_SO_4_ can be seen in Figure 6. As can be seen from Figure 6, the GCD curves of the prepared porous carbon electrodes in Na_2_SO_4_ electrolyte exhibit a symmetrical profile, which indicates that the electrodes have ideal capacitive performance and good electrochemical reversibility. At a current density of 1 A g^−1^, the specific capacitance of the YP-50F, ELC-KOH, and ELC-KOH-KCl electrodes are 98, 336, and 380 F g^−1^, respectively. The high capacitance values of ELC-KOH-KCl electrodes in the two aqueous electrolytes can be primarily attributed to the higher specific surface area and well-developed pores of ELC-KOH-KCl, which promoted the diffusion and migration of electrolyte ions in ELC-KOH-KCl. The capacitance value of the ELC-KOH-KCl electrode in KOH is higher than that in Na_2_SO_4_, a phenomenon that can be explained by the smaller size of solvated K^+^ ions compared to that of solvated Na^+^ ions (Table 2).

**Figure 7:**
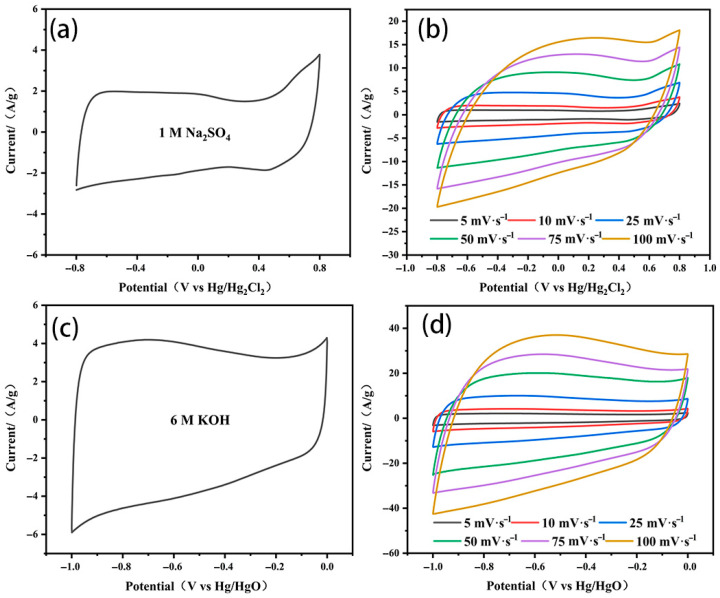
The CV curves of ELC-KOH-KCl electrode at 25 mV S^−1^ were (**a**) 1 M Na_2_SO_4_ and (**c**) 6 M KOH; CV curves of ELC-KOH-KCl electrode were (**b**) 1 M Na_2_SO_4_ and (**d**) 6 M KOH at different sweep velocities.

The capacitance behaviors of the ELC-KOH-KCl electrode in Na_2_SO_4_ and KOH aqueous electrolyte are shown in Figure 7. The CV curves of the ELC-KOH-KCl electrode at a scanning rate of 50 mV S^−1^ exhibit a well-defined rectangular shape (Figure 7a,c), which indicated that the ELC-KOH-KCl electrode had good double-layer capacitance and behavior. ELC-KOH-KCl electrodes also maintained good rectangles at sweep speeds ranging from 5 mV S^−1^ to 100 mV S^−1^ (Figure 7b,d), indicating their good magnification performance. In Na_2_SO_4_ solution, the CV curve of the ELC-KOH-KCl electrode exhibits two characteristic peaks in the voltage range of −0.8 to 0.8 V (relative to the Hg/Hg_2_Cl_2_ reference electrode), which can be attributed to the Faraday pseudocapacitance reaction involving the quinone group(C=O) [20]. When the ELC-KOH-KCl electrode was placed in a KOH electrolyte, its CV curve exhibited a significant hump at a position of approximately −1 V (relative to the Hg/HgO reference electrode) (Figure 7c), indicating that the ELC-KOH-KCl electrode also undergoes a Faraday reaction involving the carbonyl group (C=O) in the KOH environment. The carbonyl group is converted to the hydroxyl group after receiving protons and electrons, which can be represented by a specific reaction formula [21] $>{C=O+H+e}^{-}↔C-\mathrm{OH}$. Therefore, the ELC-KOH-KCl electrode in two kinds of aqueous electrolyte shows a certain pseudo-capacitance characteristic, which is attributed to the oxygen-containing functional groups on the carbon surface. However, these functional groups contribute less to capacitance than the high specific surface area (up to 2730 m^2^g^−1^) of the ELC-KOH-KCl electrode [22]. Therefore, the capacitance values of ELC-KOH-KCl electrodes in different electrolytes mainly depends on the pore structure of the ELC-KOH-KCl electrode adsorption, the capacity of ions, and the effects of ion size and steric hindrance during solvation.

**Figure 8:**
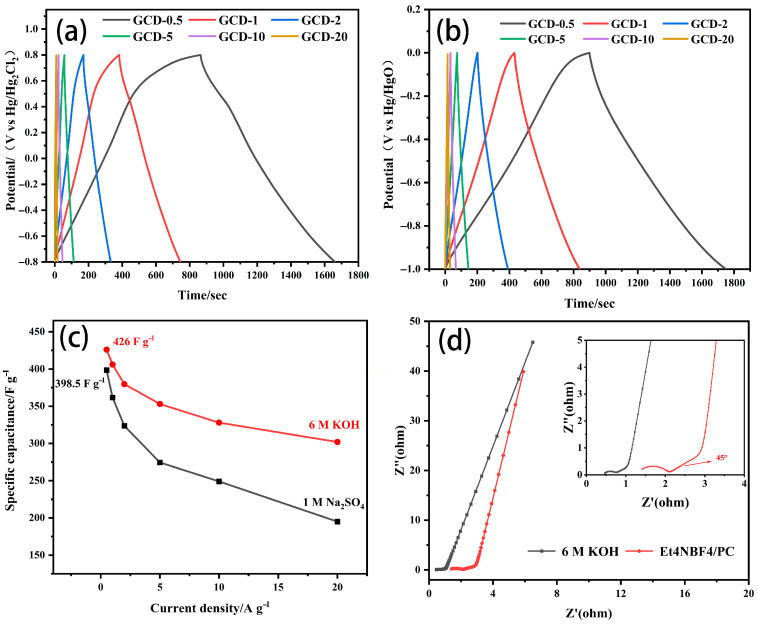
GCD curves of ELC-KOH-KCl electrodes in different aqueous electrolyte systems: (**a**) 1 M Na_2_SO_4_, (**b**) 6 M KOH, (**c**) ELC-KOH-KCl electrode-specific capacitance curves with current density changes in different aqueous electrolyte systems; (**d**) Nyquist plots of ELC electrode in different aqueous electrolytes.

**Figure 9:**
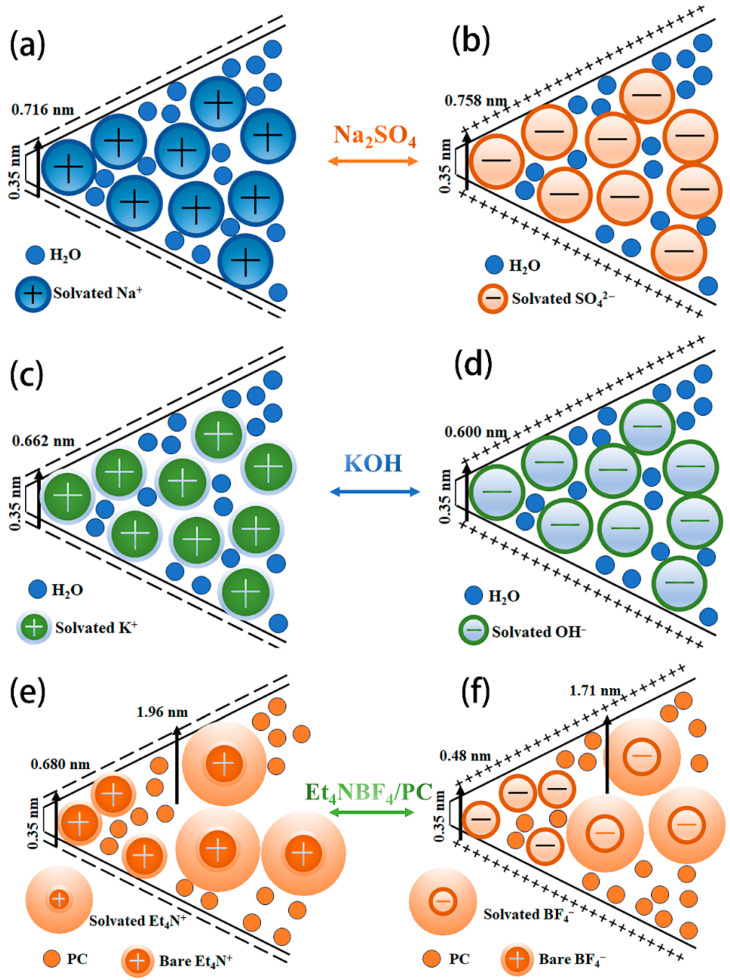
Schematic diagram of the residence of different solvated ions in micropores and small mesoporous Na_2_SO_4_ (**a**,**b**), KOH (**c**,**d**), and Et_4_NBF_4_/PC (**e**,**f**).

Figure 8a,b show the GCD curves of ELC-KOH-KCl electrodes in 1 M Na_2_SO_4_ and 6 M KOH electrolytes. The symmetry observed in all GCD curves for the ELC-KOH-KCl electrode across both aqueous electrolytes suggests optimal capacitive behavior and favorable electrochemical reversibility. At a current density of 0.5 A g^−1^, the specific capacitance of the ELC-KOH-KCl electrode in 1 M Na_2_SO_4_ and 6 M KOH was 398.5 and 426 F g^−1^, respectively (Figure 8c). The ELC-KOH-KCl electrode exhibits high specific capacitance in different aqueous electrolytes due to its high specific surface area, developed pore structure and excellent pore size distribution. These factors together promote the diffusion and transfer of electrolyte ions in ELC-KOH-KCl. The specific capacitance value of ELC-KOH-KCl in 6M KOH was higher than that in 1 M Na_2_SO_4_. This discrepancy may be attributed to the smaller size of solvated K^+^ ions hcompared to solvated Na^+^ ions (Table 2). The dissolved ion radius of Na^+^ is greater than that of K^+^, and the electrode can accommodate more K^+^ at the same pore size and specific surface area (Figure 9). Therefore, the specific capacitance of ELC-KOH-KCl electrode in 6 M KOH electrolyte is higher than that in 1 M Na_2_SO_4_. As shown in Figure 8c, the capacitance retention of the ELC-KOH-KCl electrode in Na_2_SO_4_ and KOH electrolyte was 51.5% and 70.9%, respectively, as the current density was increased from 0.5 A/g to 20 A/g, The ELC-KOH-KCl electrode demonstrates a higher capacitance retention rate in 6M KOH electrolyte, which indicates that K^+^ and OH^-^ ions exhibit a more rapid response and migration rate during the charging and discharging process at a high current density of 20 A/g. As shown in Table 2, the dissolved ion radii of K^+^ and OH^−^ are 0.385 nm and 0.300 nm, respectively. These dimensions are both small and numerically similar, which facilitates reduced steric hindrance and enhances the rates of ion response and migration within micropores. Therefore, ELC-KOH-KCl electrode exhibits superior doubling performance when utilized in a KOH environment. In contrast, the performance of the ELC-KOH-KCl electrode in a Na_2_SO_4_ electrolyte is inferior to that observed in KOH electrolyte. This discrepancy can be primarily attributed to the difference in size and charge amount of solvated Na^+^ and SO_4_^2−^ ions, which adversely affects the performance ratio in the Na_2_SO_4_ electrolyte.

The impedance characteristics of the ELC electrode in the two-electrode system were evaluated by the EIS technique. As shown in Figure 8d, the Nyquist diagrams of the ELC electrodes in different electrolytes are almost perpendicular to the real axis in the low frequency region, indicating that they all have ideal capacitance behavior [23]. In the high frequency region, the ELC electrode does not show an obvious circular arc shape in the KOH electrolyte, indicating that ion diffusion to the ELC electrode interface is very fast. KOH’s radius is less than that of Et_4_NBF_4_/PC. A typical 45° angle can be observed from the Nyquist plot of the ELC electrode in the electrolyte, indicating that the adsorption process of ions is limited by the electrolyte diffusion. The ESR value of the ELC electrode in KOH (0.29 Ω) is lower than that in Et_4_NBF_4_/PC (0.69 Ω), which is consistent with the test results. The lower the ESR value, the smaller the internal resistance of the electrode, and the order of internal resistance of the ELC electrode is as follows: KOH < Et_4_NBF_4_/PC. The results show that the conductivity of the ELC electrode in the KOH electrolyte is better than that in the Et_4_NBF_4_/PC electrolyte.

**Figure 10:**
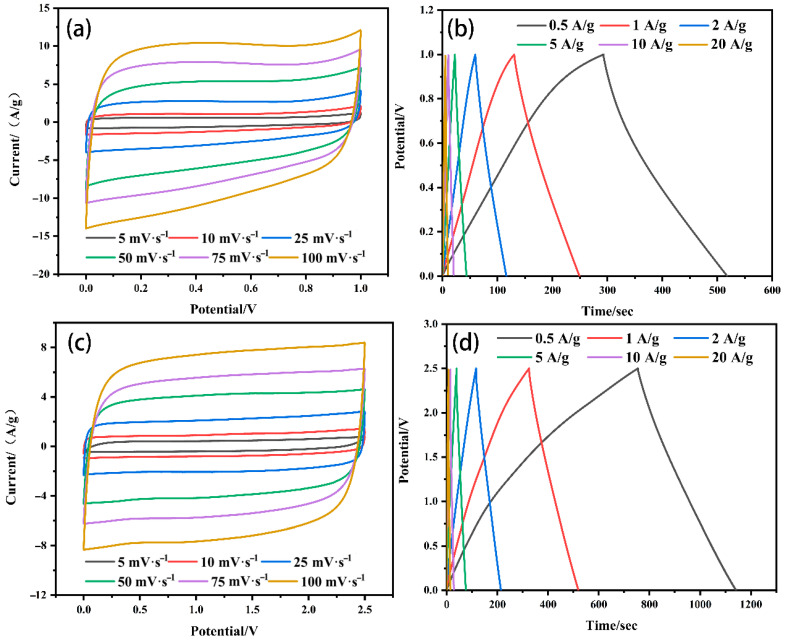
CV curves (**a**) and GCD curves (**b**) of ELC-KOH-KCl in aqueous electrolytes and CV curves (**c**) and GCD curves (**d**) in organic electrolytes.

In order to further investigate the influence of the electrolyte on the electrochemical performance of the ELC-KOH-KCl electrode, the ELC-KOH-KCl electrode was assembled into a button capacitor in the KOH and Et_4_NBF_4_/PC electrolytes. Figure 10a,c is a CV curve obtained from ELC electrodes in Et_4_NBF_4_/PC and KOH electrolytes at scanning rates ranging from 5–100 mV S^−1^, both of which exhibit a typical rectangular shape. The CV curve is more standard in the Et_4_NBF_4_/PC electrolyte. The GCD curves of the ELC electrodes in Et_4_NBF_4_/PC and KOH electrolytes are shown in Figure 10b,d. The charge–discharge curves of the ELC electrode exhibit good symmetry; even at a current density of 20 A/g, the curves maintain linear symmetry, which indicates that the ELC electrode possesses excellent reversible charge–discharge behavior [24]. In the Et_4_NBF_4_/PC electrolyte, the specific capacitance of the ELC electrode at different current densities of 0.5–20 A/g is shown in Figure 10b. At 1 A/g current density, the specific capacitance of the ELC-KOH-KCl electrode in the 6 M KOH and Et_4_NBF_4_/PC electrolyte is 249 and 192 F/g, respectively. The results indicate that the specific capacitance of the ELC-KOH-KCl electrode in the Et_4_NBF_4_/PC electrolyte is significantly lower than that in the KOH electrolyte. This discrepancy can be attributed to the radius of the dissolved ions of Et_4_N^+^ and BF_4_^−^, which are 1.96 and 1.71 nm, respectively. The micropores, which range from 0.35 to 2 nm, can accommodate only a limited number of Et_4_N^+^ and BF_4_^−^ ions. Therefore, the effective pore volume and specific surface area available for the ELC-KOH-KCl electrode in the Et_4_NBF_4_/PC electrolyte are considerably restricted. Compared with aqueous electrolytes, the ELC-KOH-KCl electrode exhibits much lower adsorption capacity for organic electrolyte ions, this reduced capacity is further exacerbated by the larger sizes of the Et_4_N^+^ and BF_4_^−^ ions, which are unable to be adsorbed within the smaller micropores. The bare ion diameters of the Et_4_N^+^ and BF_4_^−^ ions are 0.68 and 0.48 nm, respectively, and thus pore sizes smaller than these dimensions do not contribute to the capacitance. Therefore, the low specific capacitance of the ELC-KOH-KCl electrode in Et_4_NBF_4_/PC can be attributed to the inability of larger size electrolyte ions to adsorb in smaller size micropores. At a current density of 20 A/g, the capacity retention rate of the ELC-KOH-KCl electrode in Et_4_NBF_4_/PC was 81.3%, surpassing the retention rate of 75.6% observed in aqueous electrolytes. This may be because Et_4_N^+^ and BF_4_^−^ ions cannot enter the micropores, and their ion adsorption sites are mainly provided by mesoporous solutions. Compared with other electrolytes, the ion transport distance of Et_4_N^+^ and BF_4_^−^ in the electrode is greatly reduced; the adsorption/desorption process of electrolyte ions is accelerated by increasing the capacity of pores to accept electrolyte ions. A high mesoporous ratio of ELC-KOH-KCl improves the performance of the electrode at Et_4_NBF_4_/PC.

**Figure 11:**
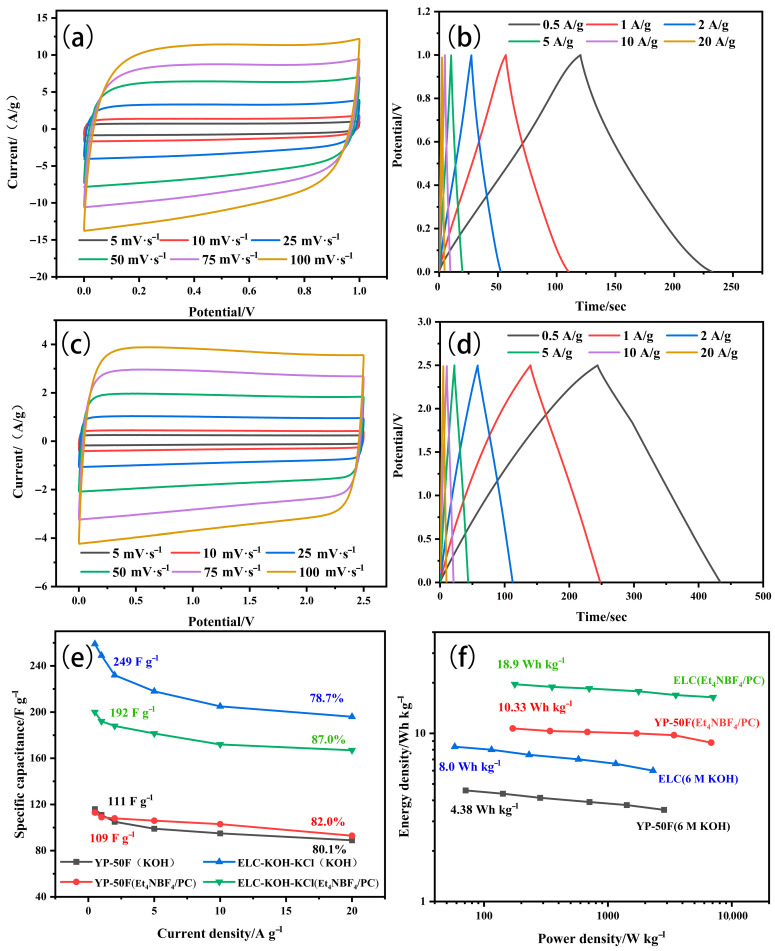
CV curves and GCD curves of YP-50F samples in aqueous electrolytes (**a**,**b**) and in organic electrolytes (**c**,**d**); volume retention rates (**e**) and Ragone plots (**f**) for each sample.

YP-50F is a kind of porous carbon material with excellent electrochemical properties for commercial supercapacitors. Therefore, YP-50F is chosen as the reference for the potential application of ELC-KOH-KCl in supercapacitors. The electrochemical properties of the YP-50F electrode in different electrolyte solutions are shown in Figure 11.

As shown in Table 3, the specific capacitance of ELC electrodes in the KOH and Et_4_NBF_4_/PC electrolytes is much higher than YP-50F, attributable to the greater specific surface area of ELC porous carbon compared to YP-50F. However, the ELC electrode has a slightly lower magnification performance than the YP-50F electrode. In organic electrolytes with large electrolyte ions (Et_4_NBF_4_/PC), the doubling performance of the ELC electrode is slightly higher than that of the YP-50F electrode. In 6M KOH alkaline electrolyte, the doubling performance of the ELC electrode is slightly lower than that of the YP-50F electrode. This is mainly because ELC porous carbon has a high mesoporous ratio (82.8%) and an excellent three-dimensional graded porous structure. The energy density of ELC in the KOH and Et_4_NBF_4_/PC electrolytes is higher than that of the YP-50F electrode, which is also in accordance with the specific capacitance of ELC in different electrolytes being higher than that of YP-50F. Therefore, ELC as a super capacitor electrode material with high specific capacitance, high energy density, and good multiplication performance, has excellent research prospects. Supercapacitors play an important role in high power demand scenarios such as electric vehicles, military equipment, industrial equipment, and electronic products. Organic electrolytes are the main electrolytes used in lithium-ion batteries. The ELC electrode has a high energy density and capacity retention in Et4NBF4PC electrolytes, which can significantly delay the capacitance decay and improve the service life of capacitors. With the development of the new energy vehicle industry, people’s requirements for the safety and environmental protection of power batteries have been improved. Because of its better safety, stability, and small impact on the environment, aqueous electrolytes have been widely used; the ionic conductivity of the ELC electrode in KOH aqueous electrolyte is much higher than that in organic electrolyte, which can greatly improve the rate and fast charging performance of the lithium-ion battery [25]. Compared to well-established organic electrolyte systems, aqueous electrolytes still have many problems to solve, such as water molecules being easily destroyed by redox, resulting in a shorter service life. At extreme temperatures, problems such as freezing or the vaporization of aqueous electrolytes can still be resolved.

## 3. Materials and Methods

### 3.1. Raw Materials, Drugs, and Instruments

Enzymatic lignin (EL), provided by Shandong Longli Bio-technology Co, Ltd.; potassium hydroxide (KOH), sodium sulfate (Na_2_SO_4_), and potassium chloride (KCl) were purchased from McLean Biological Co, Ltd. (Shanghai, China); ethanol absolute (C_2_H_6_O, 99.7%) was purchased from JiangsuStrong Functional Chemistry Co., Ltd. (Changshu, China); Et_4_NBF_4_/PC was purchased from DoDo Chemical Technology Co., Ltd. (Suzhou, China).; polytetrafluoroethylene was purchased from Aladdin Bio-chemical Technology(Shanghai, China); polytetrafluoroethylene (PTFE) was purchased from Aladdin Bio-chemical Technology Co., Ltd. (Shanghai, China); nickel foam (1.7 mm thick) was purchased from Kesheng Laboratory Equipment Co., Ltd. (Suzhou, China).; nitrogen (N_2_) was purchased from Huayang Gas Co. (Suzhou, China); and tube furnace (KJ OTF-1200×, Φ60) was purchased from KJ Materials Technology Co (Hefei, China).

### 3.2. Preparation of Lignin-Based Porous Carbon

**Figure 12:**
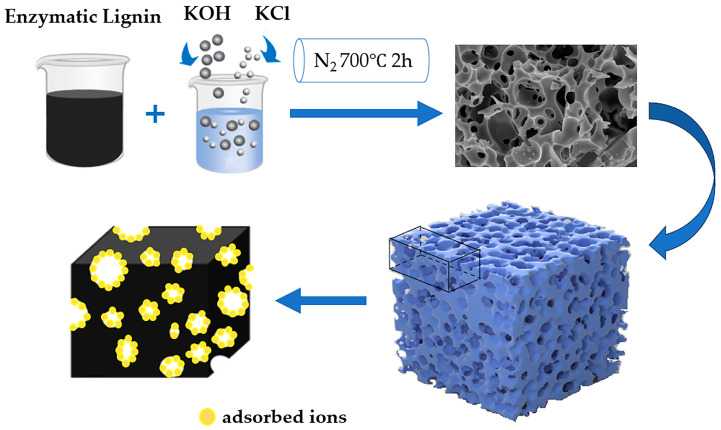
Flow chart of preparation of ELC.

EL was thoroughly combined with KOH and KCl in a ratio of 1:2:R (R = 0, 0.5) and the mixture was subsequently subjected to activation in a tubular furnace at 700 °C for 120 min under N_2_ atmosphere, with a heating rate maintained at 5 °C/min. Following activation, the resulting carbon–alkali mixture underwent a washing process, alternating between washed deionized water and 2 M HCl until the pH was neutralized to 7. After drying at 105 °C for 24 h, the enzyme-hydrolyzed lignin-based porous carbon (ELC) was obtained, designated as ELC-KOH and ELC-KOH-KCl. The process is shown in Figure 12.

YP-50F, a commercially available porous carbon, is extensively utilized in the industrial fabrication of supercapacitor electrodes due to its stability and excellent capacitance performance. To facilitate a more objective assessment of the electrochemical properties of ELC, YP-50F was used as the control sample.

### 3.3. Characterization of Lignin-Based Porous Carbon

The surface morphologies of ELC and YP-50F were observed by field-emission scanning electron microscopy (ZEISS Sigma 300, Oberkochen, Germany). To characterize the pore structure, a nitrogen adsorption/desorption measurement at 77 K was conducted in an ASAP 2460, Micromeritics Amrecian, Inc. instrument (Norcross, GA, USA). The crystal structure and microstructure of the samples were both analyzed by Raman spectroscopy (Raman, LabRAM HR Evolution, Kyoto, Japan) and XRD (X-ray diffraction, Smartlab 9, Kyoto, Japan); X-ray photoelectron spectroscopy (XPS, K-Alpha, Thermo Scientifi Inc., Waltham, MA, USA) was used in the surface analyses of the samples.

### 3.4. Characterization of Electrochemical Properties

Through an electrochemical test using an electrochemical workstation (Chenhua CHI 760E), and a three-electrode system, with 6 M KOH and 1 M Na_2_SO_4_ solution as the electrolyte, the capacitance performance of the sample was tested. The working electrode is the prepared porous carbon electrode, the opposite electrode is the platinum plate electrode, and the reference electrodes are Hg/HgO (0.098 V/NHE, 6 M KOH) and Hg/Hg_2_Cl_2_ (0.241 V/NHE, 1 M Na_2_SO_4_). The lignin carbon powder, acetylene black conductive agent, and PTFE dispersion (5% by mass fraction) were mixed at the ratio of 8:1:1, and then 99.7% anhydrous ethanol was added to grind the sample; the paint was applied to the nickel foam (active material mass ≈ 2.5 mg/cm^2^) and dried in a 70 °C vacuum dryer for 4 h. Finally, the porous carbon electrode was prepared by pressing for 10 s with 10 mpa pressure.

The method for preparing the working electrode used in 1 M Et_4_NBF_4_/PC electrolyte was mixing porous carbon with acetylene black and polyvinylidene fluoride (PVDF) at a mass ratio of 8:1:1 in an appropriate amount of deionized water. The obtained slurry was evenly coated on the copper foil and dried at 105 °C for 5 h, then cut into circular porous carbon electrodes (active material mass ≈ 2.5 mg/cm^2^) with a diameter of 14 mm. Two electrodes with similar mass of active substances were selected as positive and negative electrodes, respectively, and were assembled in a glove box filled with N_2_ in CR2032 button cell. The water and oxygen contents in the glove box were both lower than 0.1 ppm.

The cyclic voltammetry (CV) and constant current charge–discharge (GCD) of the electrodes were measured by CHI 760E electrochemical workstation. In the three-electrode system test, the mass capacitance C_g_ (F g^−1^) of the electrode was calculated by the GCD method, and the mass specific capacitance (C_S_) of the ELC electrode was calculated according to Formula (2) [26].

(1) $$C_{g}=\frac{I\times \Delta t}{m\times \Delta V}$$

(2) $$C_{g}=\frac{2I\times \Delta t}{m\times \Delta V}$$

I is the discharge current (a), ∆t is the time of discharge (s), m is the mass (g) of the active substance in a single electrode, and ∆V is the voltage change (V) in the discharge process after the IR drop.

The electric double-layer capacitor energy density E (Wh Kg^−1^) and power density P (W Kg^−1^) are calculated with Formulas (3) and (4) [13].

(3) $$E=\frac{1}{2}\cdot \frac{C_{S}}{4}{\cdot V}^{2}\frac{1}{3.6}$$

(4) $$P=\frac{E}{t}$$

where V is the potential difference (V), and T is the discharge time (h).

## 4. Conclusions

This study conducted a detailed investigation of the electrochemical properties of enzymatic hydrolyzed lignin-based porous carbon (ELC) in various electrolyte systems. By matching the size of the solvated and bare ions in the electrolytes with pore structure, the relationship between the microstructure and electrochemical properties of ELC was investigated. The high specific capacitance (249 F·g^−1^) of the ELC electrode in KOH is mainly due to the better matching of H^+^ and OH^−^ ions with the pore size of ELC. The lower specific capacitance (196 F·g^−1^) in Et_4_NBF_4_/PC electrolytes is due to the larger size and slower diffusion of Et_4_N^+^ and BF_4_^−^ ions in organic electrolytes. Despite this, the Et_4_NBF_4_/PC system possessed a high energy density (18.9 Wh kg^−1^) and excellent rate capability of 87%. These findings have implications for the potential applications of ELC in various supercapacitor systems. Specifically, for applications requiring high energy density and a long cycle life, such as energy storage systems and backup power supplies, the combination of KOH electrolytes with ELC electrodes is preferred due to their high specific capacitance. On the other hand, for applications demanding rapid charge–discharge cycles and high power output, such as energy recovery systems in electric and hybrid vehicles, the combination of Et_4_NBF_4_/PC electrolytes with ELC electrodes is more suitable, given their ability to provide high power density and excellent rate performance. By providing insights into the electrochemical performance of ELC in different electrolytes, this study offers valuable guidance for selecting electrolytes for diverse applications or industries, thereby contributing to the further development of ELC-based supercapacitor technology.

## Figures and Tables

**Table 1:** Pore structure parameters of different samples.

Sample	S_bet_/ m^2^·g^−1^	V_total_/ cm^3^·g^−1^	V_micro_ /cm^3^·g^−1^	V_meso_ /cm^3^·g^−1^	V_micro_/V_total_ (%)	V_meso_/V_total_ (%)	Dave (nm)
YP-50F	1700	0.77	0.54	0.23	70.1	29.9	1.820
ELC-KOH	1697	0.73	0.54	0.19	74.0	26.0	1.721
ELC-KOH-KCl	2730	1.45	0.25	1.20	17.2	82.8	2.123

**Table 2:** Size and ionic conductivity of bare and solvated ions.

Ion	Bare Ion Radius (nm)	Solvated Ion Radius (nm)	Ionic Conductivity (S cm^2^ mol^−1^)
Na^+^	0.095	0.358	50.1
K^+^	0.133	0.331	73.5
Et_4_N^+^	0.340	0.980	—
SO_4_^2−^	0.290	0.379	160.0
OH^−^	0.176	0.300	198.0
BF_4_^−^	0.240	0.855	—

**Table 3:** Comparison of electrochemical properties of ELC and YP-50F electrodes in different electrolyte solutions.

Electrode	Electrolyte	Specific Capacitance [F·g^−1^]	Rate Capability [%]	Energy Density [Wh·kg^−1^]
ELC	KOH	249	78.7	8.0
	Et_4_NBF_4_/PC	196	87.0	18.9
YP-50F	KOH	111	80.1	4.38
	Et_4_NBF_4_/PC	109	82.0	10.33

## Data Availability

Data are contained within the article.
